# The Association of Alcohol and Alcohol Metabolizing Gene Variants with Diabetes and Coronary Heart Disease Risk Factors in a White Population

**DOI:** 10.1371/journal.pone.0011735

**Published:** 2010-08-05

**Authors:** Lise Lotte N. Husemoen, Torben Jørgensen, Knut Borch-Johnsen, Torben Hansen, Oluf Pedersen, Allan Linneberg

**Affiliations:** 1 Research Centre for Prevention and Health, Glostrup, Denmark; 2 Faculty of Health Science, University of Copenhagen, Copenhagen, Denmark; 3 Hagedorn Research Institute and Steno Diabetes Center, Gentofte, Denmark; 4 Faculty of Health Science, University of Aarhus, Aarhus, Denmark; 5 Institute of Biomedical Sciences, University of Copenhagen, Copenhagen, Denmark; 6 Faculty of Health Science, University of Southern Denmark, Odense, Denmark; Institute of Preventive Medicine, Denmark

## Abstract

**Background:**

Epidemiological studies have shown a J- or U-shaped relation between alcohol and type 2 diabetes and coronary heart disease (CHD). The underlying mechanisms are not clear. The aim was to examine the association between alcohol intake and diabetes and intermediate CHD risk factors in relation to selected *ADH* and *ALDH* gene variants.

**Methodology/Principal Findings:**

Cross-sectional study including 6,405 Northern European men and women aged 30–60 years from the general population of Copenhagen, Denmark. Data were collected with self-administered questionnaires, a physical examination, a 2 hour oral glucose tolerance test, and various blood tests. J shaped associations were observed between alcohol and diabetes, metabolic syndrome (MS), systolic and diastolic blood pressure, triglyceride, total cholesterol, and total homocysteine. Positive associations were observed with insulin sensitivity and HDL cholesterol, and a negative association with insulin release. Only a few of the selected *ADH* and *ALDH* gene variants was observed to have an effect. The *ADH1c* (rs1693482) fast metabolizing CC genotype was associated with an increased risk of impaired glucose tolerance (IGT)/diabetes compared to the CT and TT genotypes. Significant interactions were observed between alcohol and *ADH1b* (rs1229984) with respect to LDL and between alcohol and *ALDH2* (rs886205) with respect to IGT/diabetes.

**Conclusions/Significance:**

The selected *ADH* and *ALDH* gene variants had only minor effects, and did not seem to markedly modify the health effects of alcohol drinking. The observed statistical significant associations would not be significant, if corrected for multiple testing.

## Introduction

Epidemiological studies have consistently shown that light to moderate drinkers compared to abstention are at lower risk of type 2 diabetes (T2D) and coronary heart disease (CHD) whereas heavy and excessive drinkers are at increased risk or has a risk equal to that of non-consumers [1]–[7]. The potential mechanisms of this so-called U or J-shaped association include beneficial effects on insulin sensitivity, high density lipoprotein (HDL) and low density lipoprotein (LDL) cholesterol, blood pressure and triglycerides [8]–[11]. However it has been argued that the observed inverse association between moderate alcohol consumption and diabetes and CHD is attributed to confounding factors such as a healthy lifestyle, misclassification of former alcoholics, or to constituents of alcohol other than ethanol, such as the antioxidants in grapes [12].

Alcohol is primarily metabolized in the liver. The major enzymes involved are alcohol dehydrogenase (ADH) and aldehyde dehydrogenase (ALDH). Firstly ethanol is oxidized to acetaldehyde in a reversible reaction catalyzed by the class I ADH isoenzymes (ADH1a, ADH1b, ADH1c) located in the cytosol of hepatocytes. Functional relevant polymorphisms are found in the genes encoding ADH1b and ADH1c, affecting ethanol degradation rates and alcohol intake in white populations [13]–[16]. These polymorphisms have been widely studied and related to various disease outcomes both in Asian and white populations [17]–[19]. Acetaldehyde is then oxidized to acetate and water in a non-reversible reaction catalyzed by the mitochondrial class II ALDH2 [20], [21]. The *ALDH2* gene is also polymorphic and contains an inactive variant unable to metabolize acetaldehyde resulting in the Oriental flushing syndrome [22], [23]. However, this variant is nearly absent in white populations [24]. Another less studied polymorphism in the promoter region of *ALDH2* gene has been reported in white populations, which may influence ALDH2 activity through effects on transcriptional activity [25], [26]. In addition, various single nucleotide polymorphisms (SNP's) in other ADH's and ALDH's such as the ADH7 involved in early metabolism of alcohol in the stomach mucosa and the class I ALDH1b1 (previously named as ALDHX and ALDH5) may also play a role, although their functional relevance and clinical importance is unknown [17], [27].

Variations in the alcohol metabolizing genes may help to clarify whether the association is causal, since it is less likely that an individual's genetic composition is associated with confounding factors as genotypes are distributed randomly and thus mimic a randomized trial (a principle referred to as Mendelian Randomization) [28]. Moreover a gene-environment interaction effect will only be observed if ethanol is responsible for the association.

The aim of the study was to examine the association between weekly alcohol intake and diabetes and CHD risk factors in relation to various *ADH* and *ALDH* gene variants.

## Materials and Methods

### Ethics statement

Informed written consent was obtained from all participants before participation. The study was approved by the Ethical Committees of Copenhagen and was in accordance with the principle of the Helsinki Declaration II.

### Study population

The current study is based on the Inter99 study, a population-based randomized controlled trial, investigating the effect of lifestyle intervention (smoking cessation, increased physical activity, and healthier dietary habits) on CVD. The present study was focused on the baseline data before any intervention had been offered. Data were collected with self-administered questionnaires, a physical examination, a 2 hour oral glucose tolerance test and various blood tests. Details on the study population, health examination, and the intervention program have been described elsewhere [29]. Briefly, the Inter99 study population were residents in the southern part of the former Copenhagen County. An age- and sex-stratified random sample of 13,016 men and women born in 1939–40, 1944–45, 1949–50, 1954–55, 1959–60, 1964–65, and 1969–70 was drawn from the Danish Civil Registration System and invited to participate in a health examination during 1999–2001, so that they were aged 30, 35, 40, 45, 50, 55, 60, and 65 years on the day of the examination. A total of 12,934 were eligible for invitation. The baseline participation rate was 52.5% (n = 6,784). Information on current and former nationalities of participants as well as their parents was obtained from Statistics Denmark and from the self-administered questionnaire. A Northern European origin was defined as a Danish, Norwegian, Swedish, Icelandic, or Faroese nationality. A non-Northern European origin was defined as nationalities other than the above mentioned. Both current and potential former nationalities of participants and their parents were considered. Only participants with a Northern European origin (Denmark, Norway, Sweden, Iceland, and Faroe Islands) were included in the current study (n = 6,405).

### Glucose tolerance status

All participants without known diabetes underwent a 2 hour standardized 75 g oral glucose tolerance test (OGTT) in the morning after an overnight fast. Plasma glucose, serum insulin, and serum C-peptide were measured at time (t) 0, 30, and 120 min during the OGTT. Glucose concentrations were analyzed by hexokinase/glucose-6-phosphate dehydrogenase assay (Boehringer Mannheim, Germany). Insulin and C-peptide levels were measured by a fluoroimmunoassay technique (Dako Diagnostics Ltd., UK). Glucose tolerance status was defined according to WHO diagnostic criteria 1999 [30]. Impaired fasting glucose (IFG) was defined as: fasting plasma glucose ≥6.1 mmol/l and 2 hour plasma glucose <7.8 mmol/l. Impaired glucose tolerance (IGT) was defined as: fasting plasma glucose <7.0 mmol/l, and 2 hour plasma glucose ≥7.8 mmol/l and <11.1 mmol/l. Diabetes was defined as: fasting plasma glucose ≥7.0 and 2 hour plasma glucose ≥11.1 mmol/l. IGT and diabetes were combined in the statistical analyses to increase power.

### Surrogate measures of Insulin release and insulin sensitivity

Estimates of insulin release and insulin sensitivity were estimated using both homeostasis model assessment (HOMA) based upon fasting circulating glucose and insulin levels [31].

### Alcohol

The information on alcohol drinking was obtained from the self-administered questionnaire. The on average amount and type (beer, wine, dessert wine, spirits) of alcoholic beverage consumed per week in the last 12 months were recorded. One beer, one glass of wine, or one glass of spirit was approximated to one standard drink defined as 1.5 cl or 12 g of pure ethanol. Total weekly alcohol intake was calculated as the sum of weekly intakes of beer, wine, dessert wine and spirits. For the analyses of main effects, alcohol consumption was categorized in eight categories: 0, >0–2, >2–4, >4–7, >7–14, >14–21, >21–35, >35 standard drinks per week. For the analyses of interaction effects, weekly alcohol intake was categorized in three groups: non-drinkers (0 standard drinks), light/moderate drinkers (>0–14 for women; >0–21 for men), heavy drinkers (>14 for women; >21 for men).

### ADH and ALDH gene variants

Based on previous reports on potential causal associations with disease outcomes, the following single nucleotide polymorphisms (SNPs) were examined: *ADH1b* Arg47His (rs1229984), *ADH1c* Arg271Gln (rs1693482) [15], *ADH7* (rs1573496) [17], *ALDH2* 5′-UTR A-357G (rs886205) [25], [26], [32], *ALDH1b1* Ala69Val (rs2228093) [33], [34], and *ALDH1b1* Arg90Leu (rs2073478) [33], [34]. The SNP's were genotyped by KBiosciences allele-specific PCR (KASPar) (KBiosciences, Hoddesdon, UK). All genotyping success rates were above 96.6% with a mismatch rate of 0.0% for the above mentioned SNP's in 384 duplicate samples. Rs1229984 and rs886205 deviated significantly from the Hardy-Weinberg equilibrium (p<0.001 and p = 0.025, respectively).

### Biological risk factors and metabolic syndrome

The physical examination included measurement of weight (wearing light clothes and no shoes) and height, waist circumference (in standing position at umbilical level), hip circumference, and systolic and diastolic blood pressure (measured twice in a sitting position after 5 minutes rest). Fasting triglyceride, cholesterols, homocysteine, urine albumin, and urine creatine were measured by standard techniques. Metabolic syndrome (MS) was defined according to the WHO diagnostic criteria 1999 with modifications as suggested by EGIR [30], [35]. MS was defined as insulin resistance, diabetes, impaired glucose regulation, or impaired fasting glucose in combination with two or more of the following risk factor components: dyslipidemia, hypertension, obesity or microalbuminuria. Insulin resistance was defined as fasting plasma insulin in the upper 25% quartile (≥50.0 pmol/l) of the non-diabetic population [35]. Glucose tolerance status was defined above. Dyslipidemia was defined as high triglycerides (≥1.7 mmol/l) and/or low HDL cholesterol (<0.9 mmol/l (men) and <1.0 mmol/l (women)). Hypertension was defined as high systolic blood pressure (≥140 mmHg) and/or high diastolic blood pressure (≥90 mmHg). Obesity was defined as high BMI (≥30 kg/m^2^) and/or high waist-hip ratio (>0.90 (men) and >0.85 (women)). Microalbuminuria was defined as albumin-creatinine ratio ≥30 mg/g.

### The self-administered questionnaire

The self-administered questionnaire provided information on potential confounders such as socioeconomic factors, smoking status, physical activity, general dietary habits, menopause status and use of hormone replacement therapy. Smoking status was recorded as never smokers, ex-smokers, occasional smokers (<gram tobacco per day), and daily smokers. Total physical activity was calculated on the basis of a question on commuting physical activity and a question on leisure time physical activity including walking, gardening etc. and grouped into four categories as described by von Huth et al. [36]. Based on responses to qualitative questions about intake of fruit, vegetables, fish, and saturated fat, a dietary quality score was calculated as described by Toft et al. [37]. Social class was defined on the basis of questions regarding number of years of vocational training and employment status and categorised into five classes as described previously [38]. Postmenopausal hormone replacement therapy use was recorded in three categories: 1) premenopausal, 2) postmenopausal ever user, and 3) postmenopausal never user.

### Statistical analyses

Statistics were computed with the statistical program SAS, version 9.1 (SAS Institute Inc, Cary, NC, USA). All *p* values reported are two-tailed and statistical significance was defined as *p*<0.05.

All continuous outcome variables were visually tested for approximation to the normal distribution by histograms and QQ-plots. HDL, triglyceride, homocysteine, HOMA-is, and HOMA-%B were log-transformed to achieve a normal distribution. Crude associations with continuous outcomes were examined by means and geometric means and tested for significant differences by one-way analysis of variance (F test). Crude association with dichotomous outcomes were examined in simple frequency tables and tested for significant differences by the chi-square test. Adjusted associations were evaluated in linear and logistic regression models using the PROC GLM (continuous outcomes) and the PROC GENMOD (dichotomous outcomes) procedures. Effects were reported as odds ratios (OR) and β coefficients with 95% confidence intervals (95% CI). β coefficients from models with log-transformed outcomes were back-transformed and reported as % with 95 CI. Regression models were adjusted for sex, age, BMI, dietary habits, physical activity, smoking status, socioeconomic status, and postmenopausal hormone replacement therapy use. Interaction effects were examined and evaluated in the regression models by including a product term. *F*-tests and Wald's tests for single parameters were used to test for significance in the regression analyses. Reported p values were not corrected for multiple testing. However, the large number of tests was taken into account in the interpretation of results. Persons receiving blood pressure and/or lipid lowering drugs were excluded in models including blood pressure and/or lipids. Known diabetics were excluded in analyses including HOMA estimates, since they may receive medication. Models with homocysteine included only half of the population, since only a sub-sample of the study population were a priori selected for homocysteine determination [39].

## Results

### General characteristics of the study population

The current study population consisted of 3.099 (48.4%) men and 3.306 (51.6%) women with a mean age of 46.3 (range: 29.7–61.3) years. The median weekly alcohol intake was 6.5 (range: 0–330) standard drinks including 578 (9.4%) abstainers. A total of 375 (6.2%) participants had diabetes and 1.409 (24.4%) were characterized with MS. The frequency of the *ADH* and *ALDH* minor alleles were: 0.02 (rs1229984), 0.42 (rs1693482), 0.11 (rs1573496), 0.17 (rs886205), 0.12 (rs2228093), and 0.40 (rs2073478). Further characteristics of the study population are given in table 1.

**Table 1:** Characteristics of the Inter99 study population.

Characteristic	
Men (% (n))	48.4 (3099)
Age (mean (sd))	46.26 (7.91)
Standard drinks per week (median (min, max))	6.5 (0, 330)
Abstainers (% (n))	9.4 (578)
Binge drinkers (% (n))	37.1 (2225)
Daily smoking (% (n))	35.7 (2274)
BMI≥30 kg/m^2^ (% (n))	17.6 (1124)
Very low physical activity (% (n))	12.0 (720)
Less healthy dietary habits (% (n))	16.0 (990)
Lowest social class (% (n))	3.6 (212)
Postmenopausal women (% (n))^a^	52.9 (1709)
Hormone replacement therapy (% (n))^a^	16.9 (546)

Diabetes	
Impaired glucose tolerance (% (n))	11.4 (690)
Known diabetes (% (n))	4.1 (251)
Screen-detected diabetes (% (n))	2.1 (124)
Metabolic syndrome (% (n))	24.4 (1409)
Systolic blood pressure (mean (sd))	129.31 (17.05)
Diastolic blood pressure (mean (sd))	81.77 (11.01)
Hdl cholesterol (mean (sd))	1.4 4(0.40)
Ldl cholesterol (mean (sd))	3.51 (0.96)
Total cholesterol (mean (sd))	5.54 (1.08)
Triglyceride (mean (sd))	1.33 (1.31)
Homocysteine (mean (sd))	9.00 (4.49)

Data are % (n), mean (sd), or median (min, max). N_total_ may differ due to missing information on some of the variables.

a Among women.

### Association of alcohol intake with diabetes and intermediate CHD risk factors

Alcohol consumption was significantly associated with the risk of diabetes as well as MS in a J or U shaped manner. Abstainers and excessive drinkers (>35 standard drinks per week) had the highest risks, whereas light drinkers (>2–4 standard drinks per week) had the lowest risk (table S1). Alcohol was also significantly associated with surrogate measures of insulin sensitivity and insulin release. An increasing alcohol intake was associated with increasing insulin sensitivity and decreasing insulin release when applying the HOMA model (table S1). Significant associations were also found between alcohol and all the examined biological CHD risk factors. The associations between alcohol intake and blood pressure, triglyceride, total cholesterol, and total homocysteine were J shaped, and a positive association was observed with HDL cholesterol (table S2). No significant interaction effects were observed between alcohol and sex with respect to the various outcomes (range of p values: 0.072–0.839).

### Associations of ADH and ALDH gene variants with diabetes and intermediate CHD risk factors

The *ADH1c* (rs1693482) polymorphism was significantly associated with diabetes/IGT both in crude analyses (table 2) and after adjustment for potential confounders (p = 0.011) in a co-dominant model. The fast metabolizing CC genotype were associated with an increased risk of IGT/diabetes compared to CT and TT genotypes although no clear dose-response relationship was observed (table 2). The *ADH1b* fast metabolizing AA genotype also seemed to increase the risk of IGT/diabetes compared to the GG and GA genotypes. However, only very few subjects (n = 9) were AA homozygous and the results were not statistically significant (table 2). In addition the ADH1b and ADH1c fast metabolising alleles seemed to be associated with decreased insulin sensitivity and increased insulin release (table 2). However none of the associations were statistically significant. Moreover, the *ADH1b* (rs1229984) and *ALDH1b1* (rs2073478) variants also seemed to be associated with HDL and LDL, respectively, in crude analyses (table 2, table 3), but not after adjustment for confounders (data not shown).

**Table 2:** Association between *ADH* and *ALDH* gene variants and diabetes related phenotypes.

	n	Insulin sensitivity	Insulin release	Metabolic syndrome	IGT/diabetes
		(geometric mean (95% CI))	(geometric mean (95% CI))	(% (n))	(% (n))
				n_cases_ = 1409	n_cases_ = 1065
*ADH1b* (rs1229984)					
GG, *slow*	5744	0.82 (0.81;0.83)	52.05 (51.24;53.87)	24.0 (1253)	17.1 (942)
GA	230	0.78 (0.72;0.85)	52.53 (48.64;56.74)	26.1 (55)	17.0 (36)
AA, *fast*	9	0.61 (0.33;1.10)	61.36 (34.26;109.89)		22.2 (2)
		p = 0.291	p = 0.733	p = 0.486	p = 0.92
*ADH1c* (rs1693482)					
CC, *fast*	2016	0.80 (0.80;0.83)	52.36 (50.96;53.79)	24.6 (457)	19.4 (373)
CT	2886	0.82 (0.80;0.84)	52.07 (50.94;53.22)	23.6 (614)	15.6 (428)
TT, *slow*	1031	0.83 (0.80;0.87)	51.45 (49.60;53.37)	23.8 (222)	17.3 (172)
		p = 0.229	p = 0.755	p = 0.712	P = 0.003
*ADH7* (rs1573496)					
CC	4881	0.82 (0.81;0.84)	51.98 (51.10;52.87)	24.2 (1073)	17.3 (807)
GC	1169	0.79 (0.76;0.83)	52.40 (50.62;54.24)	24.3 (260)	17.7 (198)
GG	72	0.73 (0.62;0.87)	59.30 (51.86;67.81)	25.4 (16)	16.7 (11)
		p = 0.125	P = 0.168	p = 0.969	P = 0.943
*ALDH2* (rs886205)					
TT	4075	0.82 (0.80;0.83)	52.26 (51.29;53.25)	24.6 (910)	17.6 (685)
CT	1709	0.82 (0.79;0.85)	51.55 (50.12;53.03)	23.1 (357)	16.3 (266)
CC	144	0.88 (0.79;0.98)	49.15 (44.36;54.47)	19.7 (26)	18.0 (25)
		p = 0.389	p = 0.394	p = 0.258	P = 0.512
*ALDH1b1* (rs2228093)					
CC	4586	0.82 (0.80;0.84)	51.93 (51.02;52.86)	23.9 (995)	17.2 (754)
CT	1303	0.81 (0.78;0.84)	52.74 (51.05;54.47)	24.7 (292)	17.2 (214)
TT	87	0.91 (0.80;1.04)	45.20 (40.26;50.74)	23.4 (18)	16.7 (14)
		p = 0.263	p = 0.068	p = 0.834	P = 0.992
*ALDH1b1* (rs2073478)					
TT	2142	0.82 (0.79;0.84)	52.02 (50.67;53.41)	24.7 (478)	17.7 (361)
GT	2869	0.82 (0.80;0.84)	51.93 (50.81;53.07)	23.6 (615)	16.9 (463)
GG	930	0.81 (0.77;0.84)	53.10 (51.04;55.25)	24.5 (209)	17.1 (153)
		p = 0.909	p = 0.605	p = 0.689	P = 0.746

N are the maximum number of participants in each category. N may be lower due to missing information on some variables. Data are geometric means with 95% confidence intervals (CI) or % (n). P values are F tests or Chi-square test.

**Table 3:** Association between *ADH* and *ALDH* gene variants and CHD related phenotypes.

	n	Systolic blood pressure	Diastolic blood pressure	Hdl cholesterol	Ldl cholesterol	Triglyceride	Homocysteine
		(mmHg)	(mmHg)	(mmol/l)	(mmol/l)	(mmol/l)	(µmol/l)
		(Mean (95% CI))	(Mean (95% CI))	(Geometric mean (95% CI))	(Mean (95% CI))	(Geometric mean (95% CI))	(Geometric mean (95% CI))
*ADH1b* (rs1229984)							
GG, *slow*	5744	129.3 (128.8;129.8)	81.7 (81.4;82.0)	1.39 (1.38;1.40)	3.51 (3.48;3.54)	1.12 (1.10;1.13)	8.42 (8.30;8.53)
GA	230	128.8 (126.6;131.1)	82.4 (80.8;84.0)	1.37 (1.33;1.42)	3.42 (3.29;5.54)	1.16 (1.08;1.25)	8.24 (7.77;8.74)
AA, *fast*	9	128.7 (115.8;141.5)	83.9 (77.5;90.3)	1.08 (0.92;1.26)	4.52 (2.82;4.22)	1.49 (1.05;2.12)	7.18 (4.46;11.55)
		p = 0.932	p = 0.560	p = 0.018	p = 0.382	p = 0.142	p = 0.604
*ADH1c* (rs1693482)							
CC, *fast*	2016	129.6 (128.8;130.4)	81.9 (81.4;82.4)	1.39 (1.37;1.40)	3.53 (3.48;3.57)	1.14 (1.11;1.17)	8.51 (8.33;8.70)
CT	2886	129.2 (128.6;129.9)	81.7 (81.2;82.1)	1.38 (1.37;1.40)	3.49 (3.45;3.52)	1.11 (1.09;1.13)	8.38 (8.22,8.54)
TT, *slow*	1031	128.6 (127.5;129.7)	81.7 (81.0;82.4)	1.40 (1.37;1.42)	3.51 (3.45;3.57)	1.10 (1.06;1.13)	8.29 (8.03;8.57)
		p = 0.321	p = 0.808	p = 0.624	p = 0.353	p = 0.096	p = 0.382
*ADH7* (rs1573496)							
CC	4881	129.4 (128.9;129.9)	81.9 (81.5;82.2)	1.39 (1.38;1.40)	3.51 (3.49;3.54)	1.12 (1.11;1.14)	8.35 (8.24;8.48)
GC	1169	129.3 (128.2;130.3)	81.6 (80.9;82.3)	1.39 (1.37;1.41)	3.50 3.45;3.56)	1.11 (1.07;1.14)	8.57 (8.31,8.83)
GG	72	125.7 (121.5;130.0)	79.4 (76.9;81.9)	1.38 (1.29;1.48)	3.36 (3.12;3.59)	1.12 (0.99;1.26)	9.18 (7.56;11.14)
		p = 0.227	p = 0.177	p = 0.978	p = 0.368	p = 0.669	p = 0.141
*ALDH2* (rs886205)							
TT	4075	129.5 (128.9;130.0)	81.9 (81.5;82.3)	1.39 (1.37;1.40)	3.50 (3.47;3.53)	1.12 (1.10;1.14)	8.45 (8.32;8.58)
CT	1709	129.0 (128.1;129.8)	81.4 (80.9;82.0)	1.39 (1.37;1.41)	3.53 (3.48;3.57)	1.12 (1.10;1.15)	8.31 (8.10;8.52)
CC	144	130.1 (127.0;133.3)	81.7 (79.8;83.5)	1.40 (1.33;1.46)	3.40 (3.26;3.54)	1.09 (1.00;1.20)	8.24 (7.70;8.83)
		p = 0.551	p = 0.339	p = 0.941	p = 0.298	p = 0.793	p = 0.498
*ALDH1b1* (rs2228093)							
CC	4586	129.3 (128.8;129.9)	81.8 (81.4;82.1)	1.39 (1.38;1.40)	3.51 (3.48;3.53)	1.12 (1.10;1.13)	8.44 (8.31;8.57)
CT	1303	129.4 (128.4;130.3)	81.8 (81.1;82.4)	1.38 (1.36;1.40)	3.52 (3.46;3.57)	1.12 (1.09;1.16)	8.32 (8.10;8.55)
TT	87	129.5 (125.9;133.2)	81.7 (79.4;84.0)	1.45 (1.36;1.55)	3.41 (3.20;3.62)	1.11 (0.99;1.24)	8.43 (7.41;9.59)
		p = 0.993	p = 0.999	p = 0.244	p = 0.602	p = 0.929	p = 0.684
*ALDH1b1* (rs2073478)							
TT	2142	129.4 (128.6;130.2)	81.7 (81.2;82.2)	1.38 (1.37;1.40)	3.50 (3.46;3.55)	1.12 (1.08;1.15)	8.52 (8.33;8.72)
GT	2869	129.6 (128.9;130.2)	81.9 (81.5;82.3)	1.38 (1.37;1.40)	3.54 (3.51;3.58)	1.11 (1.09;1.14)	8.39 (8.24;8.54)
GG	930	128.3 (127.1;129.5)	81.3 (80.6;82.1)	1.39 (1.36;1.41)	3.43 (3.36;3.49)	1.12 (1.08;1.16)	8.23 (7.96;8.51)
		p = 0.185	p = 0.424	p = 0.940	p = 0.007	p = 0.948	p = 0.203

N are the maximum number of participants in each category. N may be lower due to missing information on some variables. Data are geometric mean or mean with 95% confidence intervals (CI). P values are F tests.

### Interaction effects between alcohol and ADH and ALDH gene variants

In crude analyses, interaction effects were observed between alcohol and *ADH1b* (rs1229984) with respect to LDL (p_interaction_ = 0.009) and between alcohol and *ADH7* (rs1573496) with respect triglyceride (p_interaction_ = 0.021) (data not shown). However, only the interaction between alcohol and the *ADH1b* (rs1229984) variant with respect to LDL remained statistically significant after adjustment for confounders (table 4). Heavy drinking was associated with lower LDL levels among participants with the fast metabolizing A allele (table 4). Moreover, a significant interaction effect was observed between ALDH2 (rs886205) and IGT/diabetes in the adjusted model (table 5).

**Table 4:** Joint interaction effects between alcohol and ADH and ALDH gene variants with respect to CHD related phenotypes.

Genotype	Alcohol drinking	n	Systolic	Diastolic	Hdl	Ldl	Triglyceride	Homocysteine
			blood pressure	blood pressure	cholesterol	cholesterol		
			(mmHg)	(mmHg)	(mmol/l)	(mmol/l)	(mmol/l)	(µmol/l)
			(β (95% CI))	(β (95% CI))	(% (95% CI))	(β (95% CI))	(% (95% CI))	(% (95% CI))
*ADH1b* (rs1229984)								
GG (slow)	Non	493	0	0	0	0	0	0
GG (slow)	Light/moderate	4140	−0.35 (−1.99;1.29)	−0.75 (−1.81;0.32)	8.15 (5.67;10.70)	0.00 (−0.09;0.09)	−2.20 (−6.82;2.65)	−5.81 (−10.44;−0.95)
GG (slow)	Heavy	915	4.31(2.38;6.23)	1.59 (0.34;2.84)	21.93 (18.61;25.34)	−0.01 (−0.12;0.09)	3.10 (−2.66;9.19)	−3.44 (−9.10;2.58)
GA and AA (fast)	Non	37	−1.37 (−6.95;4.21)	0.29 (−3.32;3.91)	3.28 (−5.02;12.30)	0.04 (−0.29;0.37)	3.23 (−13.29;22.90)	−5.59 (−24.14;17.50)
GA and AA (fast)	Light/moderate	161	−1.16 (−4.29;1.97)	−0.42 (−2.45;1.61)	9.63 (4.88;14.58)	−0.14 (−0.31;0.04)	−3.29 (−11.80;6.04)	−7.57 (−16.19;1.94)
GA and AA (fast)	Heavy	23	1.19 (−6.01;8.39)	−1.09 (−5.75;3.58)	14.32 (3.50;26.29)	−0.69 (−1.09;−0.30)	27.68 (3.80;57.06)	−12.46 (−28.97;7.87)
			P_interaction_ = 0.836	P_interaction_ = 0.483	P_interaction_ = 0.287	P_interaction_ = 0.017	P_interaction_ = 0.132	P_interaction_ = 0.766
*ADH1c* (rs1693482)								
CC (fast)	Non	170	0	0	0	0	0	0
CC (fast)	Light/moderate	1479	0.30 (−2.41;3.00)	−0.05 (−1.80;1.71)	4.77 (0.79;8.90)	−0.03 (−0.18;0.12)	0.77 (−7.04;9.23)	−7.52 (−15.25;0.91)
CC (fast)	Heavy	282	4.16 (0.93;7.38)	2.95 (0.85;5.04)	19.02 (13.59;24.71)	−0.03 (−0.21;0.15)	3.43 (−615;13.99)	−0.88 (−10.85;10.21)
CT	Non	274	0.52 (−2.77;3.80)	0.89 (−1.25;3.02)	−2.19 (−6.66;2.49)	−0.09 (−0.28;0.09)	−1.87 (−10.97;8.15)	−4.93 (−14.19;5.34)
CT	Light/moderate	2057	−0.13 (−2.80;2.53)	−0.24 (−1.97;1.49)	5.34 (1.39;9.43)	−0.06 (−0.21;0.09)	−3.90 (−11.25;4.05)	−7.79 (−15.37;0.48)
CT	Heavy	458	5.10 (2.09;8.11)	1.87 (−0.09;3.83)	16.97 (12.00;22.15)	−0.13 (−0.30;0.04)	4.72 (−4.32;14.63)	−7.80 (−16.36;1.65)
TT (slow)	Non	85	0.55 (−3.83;4.94)	0.99 (−1.86;3.83)	−7.14 (−12.81;−1.10)	0.02 (−0.23;0.27)	1.51 (−10.97;15.74)	−4.37 (−17.02;10.22)
TT (slow)	Light/moderate	721	−0.17 (−3.01;2.68)	0.22 (−1.62;2.07)	5.31 (1.10;9.70)	−0.06 (−0.23;0.10)	−3.62 (−11.47;4.92)	−10.31 (−18.18;−1.68)
TT (slow)	Heavy	191	4.62 (1.10;8.14)	1.81 (−0.48;4.09)	19.30 (13.44;25.46)	−0.03 (−0.23;0.17)	2.05 (−8.09;13.31)	−10.67 (−20.38;0.22)
			P_interaction_ = 0.878	P_interaction_ = 0.566	P_interaction_ = 0.131	P_interaction_ = 0.692	P_interaction_ = 0.619	P_interaction_ = 0.515
*ADH7* (rs1573496)								
CC	Non	421	0	0	0	0	0	0
CC	Light/moderate	3507	−0.13 (−1.92;1.57)	−0.33 (−1.50;0.83)	6.54 (3.87;9.27)	−0.02 (−0.12;0.08)	−2.03 (−7.08;3.29)	−7.07 (−11.98;−1.88)
CC	Heavy	781	4.43 (2.33;6.54)	2.15 (0.78;3.51)	19.58 (16.05;23.22)	−0.02 (−0.13;0.10)	5.85 (−0.56;12.68)	−4.15 (−10.17;2.28)
GC and GG	Non	121	−0.76 (−4.20;2.68)	−0.05 (−2.28;2.18)	−0.87 (−5.61:4–10)	−0.07 (−0.26;0.13)	−4.73 (−13.98;5.52)	−1.38 (−11.64;10.06)
GC and GG	Light/moderate	878	−0.43 (−2.47;1.61)	−0.72 (−2.04;0.60)	6.47 (3.44;9.60)	0.01 (−0.11;0.12)	−1.41 (−7.18;4.72)	−1.89 (−7.79;4.39)
GC and GG	Heavy	195	4.82 (1.90;7.75)	0.87 (−1.03;2.77)	20.49 (15.49;25.69)	−0.11 (−0.27;0.06)	−0.77 (−9.16;8.40)	−3.94 (−12.70;5.70)
			P_interaction_ = 0.853	P_interaction_ = 0.597	P_interaction_ = 0.872	P_interaction_ = 0.314	P_interaction_ = 0.219	P_interaction_ = 0.344
*ALDH2* (rs886205)								
TT	Non	345	0	0	0	0	0	0
TT	Light/moderate	2953	0.40 (−1.54;2.35)	−0.59 (−1.84;0.67)	7.45 (4.54;10.45)	−0.03 (−0.13;0.08)	−3.39 (−8.78;2.31)	−5.21 (−10.74;0.66)
TT	Heavy	644	5.63 (3.35;7.91)	1.79 (0.31;3.26)	20.57 (16.72;24.55)	−0.08 (−0.20;0.05)	0.76 (−5.81;7.84)	−3.07 (−9.68;4.04)
CT and CC	Non	179	2.34 (−0.82;5.50)	0.01 (−2.03;2.06)	−0.78 (−5.12;3.75)	−0.08 (−0.26;0.10)	−4.81 (−13.27;4.47)	2.32 (−7.12;12.73)
CT and CC	Light/moderate	1305	0.40 (−1.65;2.46)	−0.98 (−2.31;0.35)	8.41 (5.29;11.63)	−0.01 (−0.13;0.11)	−4.63 (−10.26;1.36)	−5.37 (−11.22;0.86)
CT and CC	Heavy	287	3.55 (0.84;6.27)	1.05 (−0.70;2.81)	22.20 (17.52;27.06)	0.00 (−0.16;0.15)	−4.63 (−10.26;1.36)	−2.45 (−10.90;6.81)
			P_interaction_ = 0.080	P_interaction_ = 0.843	P_interaction_ = 0.743	P_interaction_ = 0.412	P_interaction_ = 0.224	P_interaction_ = 0.889
*ALDH1b1* (rs2228093)								
CC	Non	411	0	0	0	0	0	0
CC	Light/moderate	3290	−0.19 (−2.00;1.63)	−0.68 (−1.85;0.50)	7.67 (4.95;10.46)	0.02 (−0.08;0.12)	−5.14 (−10.06;0.05)	−3.57 (−8.90;2.08)
CC	Heavy	715	3.48 (1.34;5.63)	1.20 (−0.19;2.59)	21.02 (17.38;24.78)	−0.02 (−0.14;0.10)	−0.04 (−6.20;6.52)	−2.26 (−8.71;4.64)
CT and TT	Non	114	−0.01 (−3.57;3.56)	−0.32 (−2.63;1.99)	−2.45 (−7.30;2.64)	0.09 (−0.11;0.29)	−8.13 (−17.36;2.14)	5.26 (−5.24;16.93)
CT and TT	Light/moderate	1003	−0.84 (−2.85;1.18)	−0.99 (−2.29;0.31)	6.95 (3.94;10.04)	0.00 (−0.11;0.12)	−3.09 (−8.68;2.83)	−4.95 (−10.73;1.21)
CT and TT	Heavy	225	6.12 (3.34;8.90)	2.17 (0.37;3.97)	20.56 (15.81;25.51)	0.01 (−0.15;0.16)	5.49 (−2.97;14.69)	−0.23 (−8.76;9.09)
			P_interaction_ = 0.066	P_interaction_ = 0.364	P_interaction_ = 0.782	P_interaction_ = 0.570	P_interaction_ = 0.105	P_interaction_ = 0.423
*ALDH1b1* (rs2073478)								
TT	Non	190	0	0	0	0	0	0
TT	Light/moderate	1545	−0.18 (−2.81;2.46)	−1.20 (−2.90;0.50)	5.96 (2.06;10.02)	0.00 (−0.15;0.15)	−1.91 (−9.28;6.07)	−3.78 (−11.34;4.42)
TT	Heavy	326	3.22 (0.10;6.35)	0.95 (−1.07;2.98)	18.97 (13.76;24.42)	−0.08 (−0.26;0.10)	0.83 (−8.14;10.67)	−2.80 (−11.83;7.15)
GT	Non	254	0.41 (−2.85;3.68)	0.16 (−1.95;2.27)	−2.58 (−6.97;2.02)	0.00 (−0.18;0.19)	0.05 (−9.11;10.13)	−1.41 (−10.78;8.95)
GT	Light/moderate	2066	−0.37 (−2.96;2.23)	−0.84 (−2.52;0.84)	6.39 (2.52;10.41)	0.02 (−0.12;0.17	−3.89 (−11.02;3.82)	−6.72 (−13.95;1.11)
GT	Heavy	442	4.95 (2.01;7.90)	1.54 (−0.37;3.44)	18.79 (13.87;23.93)	−0.01 (−0.18;0.16)	5.82 (−3.10;15.57)	−4.68 (−13.24;4.74)
GG	Non	85	−1.52 (−5.93;2.89)	−1.81 (−4.66;1.05)	−1.90 (−7.95;4.55)	−0.05 (−0.30;0.20)	−3.89 (−15.82;9.71)	2.71 (−10.68;18.10)
GG	Light/moderate	656	−1.14 (−3.95;1.67)	−1.18 (−3.00;0.63)	5.71 (1.54;10.04)	−0.08 (−0.24;0.07)	−0.15 (−8.16;8.56)	−9.13 (−16.74;−0.82)
GG	Heavy	161	3.87 (0.22;7.52)	0.75 (−1.61;3.12)	21.53 (15.34;28.04)	−0.02 (−0.23;0.18)	0.86 (−9.52;12.43)	−5.22 (−15.30;6.06)
			P_interaction_ = 0.673	P_interaction_ = 0.804	P_interaction_ = 0.593	P_interaction_ = 0.745	P_interaction_ = 0.282	P_interaction_ = 0.837

N are the maximum number of participants in each category. N may be lower due to missing information on some variables. Data are β coefficients with 95% confidence intervals (CI) from adjusted regression analyses. The category “wildtype non drinkers” was set as the joint reference group. β coefficients from models with log-transformed outcomes were back-transformed and reported as % with 95% CI. All p values are F tests.

**Table 5:** Joint interaction effects between alcohol and *ADH* and *ALDH* gene variants with respect to diabetes related phenotypes.

Genotype	Alcohol drinking	n	Insulin sensitivity	Insulin release	Metabolic syndrome	IGT/diabetes
			(% (95% CI))	(% (95% CI))	(OR (95% CI))	(OR (95% CI))
					n_cases_ = 1409	n_cases_ = 1065
ADH1b (rs1229984)						
GG (slow)	Non	493	0	0	1	1
GG (slow)	Light/moderate	4140	13.11 (6.67;19.94)	−12.66 (−17.42;−7.63)	0.63 (0.47;0.85)	0.67 (0.50;0.88)
GG (slow)	Heavy	915	22.66 (14.45;31.47)	−25.28 (−30.06;−20.17)	0.93 (0.66;1.31)	1.05 (0.76;1.45)
GA and AA (fast)	Non	37	−11.60 (−27.68;8.04)	2.35 (−15.48;23.95)	1.31 (0.48;3.56)	0.76 (0.26;2.22)
GA and AA (fast)	Light/moderate	161	14.55 (2.39;28.16)	−14.14 (−22.86;−4.43)	0.60 (0.33;1.08)	0.63 (0.35;1.13)
GA and AA (fast)	Heavy	23	8.74 (−14.75;38.72)	−13.94 (−31.78;8.55)	0.35 (0.08;1.45)	0.90 (0.28;2.91)
			P_interaction_ = 0.353	P_interaction_ = 0.449	P_interaction_ = 0.326	P_interaction_ = 0.929
ADH1c (rs1693482)						
CC (fast)	Non	170	0	0	1	1
CC (fast)	Light/moderate	1479	10.37 (0.14;21.66)	−8.43 (−16.56;0.49)	0.76 (0.46;1.26)	0.75 (0.48;1.19)
CC (fast)	Heavy	282	20.06 (6,84;34.91)	−20.32 (−28.78;−10.93)	1.21 (0.67;2.18)	1.08 (0.64;1.85)
CT	Non	274	−3.42 (−14.16;8.66)	7.40 (−4.03;20.18)	1.45 (0.80;2.63)	0.95 (0.55;1.64)
CT	Light/moderate	2057	14.44 (3.97;25.96)	−9.50 (−17.43;−0.80)	0.72 (0.44;1.19)	0.53 (0.34;0.83)
CT	Heavy	458	21.83 (9.28;35.81)	−21.31 (−29.07;−12.71)	1.04 (0.60;1.80)	1.02 (0.62;1.69)
TT (slow)	Non	85	−0.37 (−15.01;16.81)	3.77 (−10.83;20.76)	1.11 (0.51;2.44)	0.65 (0.29;1.43)
TT (slow)	Light/moderate	721	13.78 (2.69;26.06)	−9.77 (−18.19;−0.48)	0.84 (0.49;1.42)	0.70 (0.43;1.15)
TT (slow)	Heavy	191	22.27 (7.67;38.85)	−24.79 (−33.38;−15.08)	1.10 (0.59;2.07)	0.94 (0.53;1.68)
			P_interaction_ = 0.855	P_interaction_ = 0.632	P_interaction_ = 0.614	P_interaction_ = 0.315
*ADH7* (rs1573496)						
CC	Non	421	0	0	1	1
CC	Light/moderate	3507	13.56 (6.55;21.04)	−12.42 (−17.59;−6.92)	0.63 (0.46;0.88)	0.78 (0.57;1.06)
CC	Heavy	781	20.02 (11.34;29.38)	−23.08 (−28.41;−17.36)	1.03 (0.71;1.50)	1.19 (0.83;1.70)
GC and GG	Non	121	−2.80 (−14.07;9.95)	1.59 (−9.68;14.28)	1.07 (0.58;1.97)	1.46 (0.83;2.56)
GC and GG	Light/moderate	878	9.94 (2.26;18.20)	−10.36 (−16.35;−3.93)	0.72 (0.50;1.05)	0.82 (0.57;1.18)
GC and GG	Heavy	195	23.49 (11.10;37.26)	−28.41 (−35.28;−20.80)	0.74 (0.44;1.26)	1.25 (0.77;2.02)
			P_interaction_ = 0.526	P_interaction_ = 0.180	P_interaction_ = 0.227	P_interaction_ = 0.577
*ALDH2* (rs886205)						
TT	Non	345	0	0	1	1
TT	Light/moderate	2953	18.59 (10.67;27.06)	−14.73 (−20.15;−8.94)	0.53 (0.37;0.74)	0.54 (0.39;0.74)
TT	Heavy	644	25.56 (15.76;36.18)	−25.56 (−31.09;−19.59)	0.78 (0.52;1.16)	0.83 (0.58;1.20)
CT and CC	Non	179	7.60 (−3.86;20.44)	−4.99 (−14.67;5.79)	0.59 (0.33;1.05)	0.43 (0.24;0.76)
CT and CC	Light/moderate	1305	17.35 (9.06;26.27)	−14.79 (−20.52;−8.64)	0.43 (0.30;0.62)	0.49 (0.35;0.69)
CT and CC	Heavy	287	31.78 (19.56;45.25)	−29.09 (−35.36;−22.22)	0.62 (0.38;1.00)	0.79 (0.51;1.22)
			P_interaction_ = 0.224	P_interaction_ = 0.440	P_interaction_ = 0.591	P_interaction_ = 0.038
*ALDH1b1* (rs2228093)						
CC	Non	411	0	0	1	1
CC	Light/moderate	3290	15.14 (7.98;22.78)	−13.69 (−18.81;−8.23)	0.59 (0.42;0.81)	0.63 (0.46;0.86)
CC	Heavy	715	24.91 (15.71;34.84)	−25.64 (−30.87;−20.02)	0.81 (0.55;1.19)	0.99 (0.70;1.42)
CT and TT	Non	114	0.79 (−11.56;14.86)	−4.00 (−15.24;8.73)	1.06 (0.56;2.01)	0.84 (0.45;1.57)
CT and TT	Light/moderate	1003	13.48 (5.66;21.87)	−12.88 (−18.61;−6.74)	0.67 (0.46;0.97)	0.84 (0.45;1.57)
CT and TT	Heavy	225	19.21 (7.81 (31.81)	−25.16 (−31.99;−17.64)	1.14 (0.70;1.85)	1.11 (0.70;1.75)
			P_interaction_ = 0.756	P_interaction_ = 0.754	P_interaction_ = 0.673	P_interaction_ = 0.699
*ALDH1b1* (rs2073478)						
TT	Non	190	0	0	1	1
TT	Light/moderate	1545	16.98 (6.57;28.41)	−15.55 (−22.75;−7.67)	0.56 (0.35;0.88)	0.75 (0.48;1.19)
TT	Heavy	326	29.77 (16.08;45.07)	−30.03 (−37.10;−22.16)	0.77 (0.45;1.32)	1.03 (0.61;1.74)
GT	Non	254	1.17 (−9.87;13.57)	−2.45 (−12.65;8.94)	0.86 (0.48;1.53)	0.98 (0.56;1.71)
GT	Light/moderate	2066	16.75 (6.50;27.98)	−15.18 (−22.31;−7.38)	0.54 (0.35;0.85)	0.67 (0.43;1.06)
GT	Heavy	442	22.26 (10.09;35.77)	−25.49 (−32.60;−17.64)	0.86 (0.48;1.53)	1.11 (0.67;1.83)
GG	Non	85	5.49 (−9.94;23.57)	−6.90 (−19.95;8.29)	0.94 (0.44;2.02)	1.40 (0.68;2.86)
GG	Light/moderate	656	11.93 (1.33;23.64)	−12.45 (−20.40;−3.71)	0.64 (0.39;1.05)	0.77 (0.47;1.26)
GG	Heavy	161	22.61 (7.69;39.59)	−24.27 (−33.10;−14.27)	0.72 (0.38;1.37)	1.32 (0.72;2.40)
			P_interaction_ = 0.574	P_interaction_ = 0.460	P_interaction_ = 0.745	P_interaction_ = 0.855

N are the maximum number of participants in each category. N may be lower due to missing information on some variables. Data are β coefficients or odds ratios (OR) with 95% confidence intervals (CI) from adjusted regression analyses. The category “wildtype non drinkers” was set as the joint reference group. β coefficients from models with log-transformed outcomes were back-transformed and reported as % with 95% CI. All p values are F tests.

## Discussion

In this study we examined the association between alcohol and diabetes and intermediate CHD risk factors in relation to selected *ADH* and *ALDH* gene variants in an adult general population sample. We observed a strong association between alcohol intake and diabetes, MS and several CHD risk factors. The *ADH* and *ALDH* gene variants on the other hand had only minor effects, and did not seem to markedly modify the health effects of alcohol drinking.

Our results confirm previous studies showing a U- or J-shaped relation between alcohol and type 2 diabetes and CHD [1]–[7]. Meta analyses have shown that light-moderate alcohol consumption is associated with a protective effect in the order of 30–40% with respect to type 2 diabetes and 20–30% with respect to CHD [2], [6], [7], [40].

The finding of positive association with surrogate measures of insulin sensitivity and a negative relation with insulin release, suggest that the J-shaped relation may be explained by a beneficial effect of moderate alcohol intake on the insulin sensitivity and an adverse effect of heavy alcohol intake on insulin release perhaps caused by a toxic alcohol effect on the pancreatic β cells. However the insulin release may also decrease with increasing alcohol intake due to lower demands caused by the increasing sensitivity. In this context, misclassification of alcohol exposure should also be considered (see below).

Furthermore, our results support previous findings of beneficial effects of alcohol drinking on insulin sensitivity and HDL cholesterol levels [9]–[11]. Also in accordance with our results elevated blood pressure, triglyceride, total and LDL cholesterol in heavy-excessive drinkers have been reported previously [8], [9]. Studies on the relationship between total alcohol intake and circulating homocysteine levels have been inconsistent, but several studies have shown a lowering effect of beer drinking on plasma homocysteine concentrations, which has also been reported previously for this cohort [38].

In the current study we observed significant associations between *ADH1c* (rs1693482) and IGT/diabetes (co-dominant model). The fast metabolizing C allele was related to a higher risk of IGT/diabetes. This is supportive of a protective role of alcohol, since individuals with genotypes coding for fast alcohol degradation have lower blood alcohol concentrations. Moreover, the *ADH1c* C allele has been associated with a lower alcohol intake also contributing to lower blood alcohol concentrations [13], [14]. Beulens et al. also reported in a nested case-control study of 1.023 white men and women with incident diabetes and 1.382 controls that the *ADH1c* genotype modified the association between alcohol consumption and diabetes. In this study the slow metabolizing allele seemed to attenuate the lower risk of diabetes among moderate to heavy drinkers [41]. Although we did observe an association between the *ADH1c* (rs1693482) variant and IGT/diabetes as well as insulin sensitivity, the direction was opposite, and we could not confirm the results. However in accordance with our results, Hines et al. showed in a nested case-control study of 1166 U.S. male physicians (396 patients and 777 controls) that the slow oxidizing *ADH1c* allele is associated with reduced risk of myocardial infarct in moderate drinkers [42]. This has been confirmed in other populations [43], [44]. In addition an interaction between *ADH1c* and the level of alcohol consumption in relation to HDL has been reported in several studies [42], [45] although not in all [43], [46].

The *ADH1b* rs1229984 GG slow genotype has been associated with elevated blood pressure, triglycerides, and uric acid in one study of Japanese [47], whereas another study on Europeans found no relation to HDL [46]. We observed a decreased fasting serum LDL level among heavy drinkers with the intermediate/fast *ADH1b* (rs1229984) GA/AA genotype.

An interaction effect was also observed between *ALDH2* (rs886205) and IGT/diabetes in the current study. The relationship of this *ALDH2* variant as well as the *ADH7* and *ALDH1b1* variants with diabetes and CHD related phenotypes have not been studied previously, except from a previous study (n = 1,216) from our group [13]. In this study we observed an association between the *ALDH1B1* (rs2228093) gene variant and diastolic blood pressure, which was not confirmed in the current study. Besides that we did not observe any effects of *ADH1b* (rs1229984), *ADH1c* (rs1693482), *ALDH1b1* (rs2228093, rs2073478), and *ALDH2* (rs886205) with respect to blood pressure, cholesterols, and triglyceride in this previous study [13]. Studies on the inactive *ALDH2* Asian variant have found no association with neither cholesterols [48] nor blood pressure [49], [50].

Taken as a whole the studies on the effects of genetic variation in *ADH* and *ALDH* on the risk of type 2 diabetes and CHD have been inconsistent. One explanation could be that drinking patterns and levels of intake differ between Danes and e.g. the US population. Moreover, our results with the genetic variants would not be significant after correction for multiple testing, and we believe that many of the inconsistencies between studies may be due to chance findings, although we cannot exclude that they are real. Nonetheless, these results do not exclude a causal relationship between alcohol and diabetes and CHD, but they do suggest that the influence of genetic variation in the alcohol metabolizing enzymes is relatively small.

Previously, we showed that the *ADH1b* (rs1229984), *ADH1c* (rs1693482) and *ALDH1b1* (rs2228093) genotypes is associated with amount of alcohol intake, which may have interfered with the principles of Mendelian randomization and influenced the results. Individuals with *ADH1b* and *ADH1c* slow metabolizing genotypes were drinking more [51]. Thus *ADH1b* and *ADH1c* slow metabolizers have higher blood ethanol concentrations due to both the lower activity of the enzyme and to a higher alcohol intake. Both effects stem directly from the genotype and cannot be separated. If ethanol is responsible for the adverse/beneficial effects of alcohol drinking the observed associations would have been intensified. However, if a more downstream metabolite e.g. acetaldehyde is responsible for the effects of alcohol, the observed associations may have been attenuated towards the null value due to opposing effects of enzyme activity and alcohol intake (low ADH enzyme activity results in low acetaldehyde peak levels and a high alcohol intake results in high acetaldehyde levels). The *ALDH1b1* (rs2228093) genotypes have also been shown to influence alcohol drinking habits, but the effects of this polymorphism on enzyme activity is unknown.

Several other potential limitations of the study should be considered. Firstly, despite the relatively large number of participants in the current study, it is possible that the study may have missed important effects of the genetic variants due to low statistical power. In contrast, a large number of statistical tests have been performed in this study which increases the risk of chance findings. The observed statistical significant associations involving the *ADH* and *ALDH* gene variants would not be significant after correction for multiple testing. Moreover, rs1229984 and rs886205 were not in Hardy-Weinberg equilibrium. However the prevalence of the gene variants was similar to other studies on European populations, suggesting that this has perhaps happened by chance [14], [26], [32], [34], [52]. Also, among 384 replicate samples, we found no genotype errors for the two SNPs. In addition, the alcohol intake was estimated on the basis of a self-administered questionnaire and not by an objective method. Due to social desirability bias the participants may have underreported their actual intake. However, the ranking of participants were probably quite accurate, as total weekly alcohol intake as assessed by this method in another population-based study has previously been found to be positively associated with markers of high alcohol intake [13], [53]. However, the J-shape might also be explained by the possibility that some previous or current heavy drinkers are misclassified as non-drinkers. Finally, the cross-sectional study design may not allow us to draw firm conclusions about the causal direction of associations between alcohol and e.g. diabetes. Thus, we cannot exclude the possibility that persons with diabetes have changed their alcohol intake due to the disease. However, associations with genetic variants may favour a causal relationship, since an individual's genetic composition does not change over time and is less likely to be associated with confounding factors.

In conclusion, strong associations between weekly alcohol intake and diabetes, MS and several intermediate CHD risk factors were observed. The *ADH* and *ALDH* gene variants on the other hand had only minor effects, and did not seem to modify the health effects of alcohol drinking greatly in this study.

## Supporting Information

Table S2 Associations between weekly alcohol intake and CHD related phenotypes.
(0.06 MB DOC)

Table S1 Associations between weekly alcohol intake and diabetes related phenotypes.
(0.05 MB DOC)
