# Supplementary material for: The Association of Alcohol and Alcohol Metabolizing Gene Variants with Diabetes and Coronary Heart Disease Risk Factors in a White Population
Source: PLoS One. 2010 Aug 5;5(8):e11735. doi: 10.1371/journal.pone.0011735 (PMC2916825; doi:10.1371/journal.pone.0011735)
Supplement: Table S2 — Associations between weekly alcohol intake and CHD related phenotypes. (0.06 MB DOC) [file pone.0011735.s001.doc]

|  | Usual weekly alcohol intake during the last 12 months (standard drinks) | | | | | | | |  |
| --- | --- | --- | --- | --- | --- | --- | --- | --- | --- |
|  | 0 | >0-2 | >2-4 | >4-7 | >7-14 | >14-21 | >21-35 | >35 | p |
|  | n=578 | n=1010 | n=835 | n=929 | n=1339 | n=702 | n=520 | n=259 |  |
| Insulin sensitivity (HOMA-is) | -7.40 | 0 (ref.) | 6.15 | 5.92 | 6.85 | 10.41 | 14.57 | 8.37 |  |
| Adjusted regression (% (95% CI)) | (-13.05;-1.39) |  | (0.33;12.31) | (0.31;11.86) | (1.55;12.43) | (3.98;17.23) | (7.11;22.54) | (18.33;0.07) | <0.001 |
| Insulin release (HOMA-%B) | 6.90 | 0 (ref.) | -4.15 | -7.36 | -11.82 | -14.92 | -20.67 | -20.60 |  |
| Adjusted regression (% (95% CI)) | (0.67;13.51) |  | (-9.17;1.14) | (-12.05:-2.43) | (-15.99;-7.43) | (-19.65;-9.92) | (-25.59;-15.41) | (-26.98;-13.66) | <0.001 |
| Diabetes | 1.62 | 1 (ref.) | 0.59 | 0.78 | 0.69 | 0.96 | 0.83 | 1.59 |  |
| Adjusted regression (OR (95% CI)) | (1.01;2.60) |  | (0.34;1.02) | (0.48;1.27) | (0.44;1.09) | (0.59;1.57) | (0.49;1.43) | (0.90;2.80) | 0.001 |
| Metabolic syndrome | 1.48 | 1 (ref.) | 0.82 | 0.83 | 0.89 | 1.03 | 1.28 | 1.47 |  |
| Adjusted regression (OR (95% CI)) | (1.06;2.05) |  | (0.60;1.12) | (0.61;1.13) | (0.67;1.18) | (0.75;1.42) | (0.92;1.18) | (0.97;2.21) | 0.001 |
| IGT/diabetes | 1.16 | 1 (ref.) | 0.64 | 0.79 | 0.72 | 0.91 | 1.12 | 1.83 |  |
| Adjusted regression (OR (95% CI)) | (0.86;1.58) |  | (0.47;0.87) | (0.59;1.05) | (0.55;0.94) | (0.67;1.23) | (0.82;1.55) | (1.25;2.68) | <0.001 |

Data are odds ratios (OR) or β coefficients with 95% confidence intervals (CI) from adjusted regression analyses. β coefficients from models with log-transformed outcomes were back-transformed and reported as % with 95% CI. P values are F tests.
