# Supplementary material for: The Association of Alcohol and Alcohol Metabolizing Gene Variants with Diabetes and Coronary Heart Disease Risk Factors in a White Population
Source: PLoS One. 2010 Aug 5;5(8):e11735. doi: 10.1371/journal.pone.0011735 (PMC2916825; doi:10.1371/journal.pone.0011735)
Supplement: Table S1 — Associations between weekly alcohol intake and diabetes related phenotypes. (0.05 MB DOC) [file pone.0011735.s002.doc]

|  | Usual weekly alcohol intake during the last 12 months (standard drinks) | | | | | | | |  |
| --- | --- | --- | --- | --- | --- | --- | --- | --- | --- |
|  | 0 | >0-2 | >2-4 | >4-7 | >7-14 | >14-21 | >21-35 | >35 | p |
|  | n=578 | n=1010 | n=835 | n=929 | n=1339 | n=702 | n=520 | n=259 |  |
| Systolic blood pressure (mmHg) | 0.10 | 0 (ref.) | -0.34 | 0.26 | 0.07 | 1.13 | 4.15 | 7.30 |  |
| Adjusted regression (β (95% CI)) | (-1.66;1.86) |  | (-1.91;1.23) | (-1.25;1.77) | (-1.33;1.48) | (-0.54;2.80) | (2.29;6.02) | (4.87;9.72) | p<0.001 |
| Diastolic blood pressure (mmHg) | 0.25 | 0 (ref.) | -0.91 | -0.61 | -0.20 | 0.55 | 1.65 | 3.65 |  |
| Adjusted regression (β (95% CI)) | (-0.89;1.39) |  | (-1.92;0.11) | (-1.59;0.37) | (-1.11;0.71) | (-0.54;1.63) | (0.44;2.86) | (2.07;5.23) | p<0.001 |
| Hdl cholesterol (mmol/l) | -1.50 | 0 (ref.) | 4.23 | 6.92 | 10.95 | 14.21 | 20.36 | 26.38 |  |
| Adjusted regression (% (95% CI)) | (-3.91;0.97) |  | (1.93;6.58) | (4.64;9.25) | (8.74;13.20) | (11.52;16.96) | (17.18;23.63) | (22.07;30.85) | p<0.001 |
| Ldl cholesterol (mmol/l) | -0.01 | 0 (ref.) | -0.03 | 0.01 | -0.07 | 0.04 | 0.02 | -0.21 |  |
| Adjusted regression (β (95% CI)) | (-0.10;0.09) |  | (-0.12;0.06) | (-0.07;0.10) | (-0.16;0.01) | (-0.05;0.14) | (-0.09;0.13) | (-0.35;-0.07) | p=0.007 |
| Total cholesterol (mmol/l) | -0.04 | 0 (ref.) | -0.02 | 0.07 | 0.05 | 0.22 | 0.31 | 0.20 |  |
| Adjusted regression (β (95% CI)) | (-0.15;0.07) |  | (-0.11;0.08) | (-0.03;0.16) | (-0.04;0.14) | (0.11;0.33) | (0.19;0.43) | (0.04;0.35) | p<0.001 |
| Triglyceride (mmol/l) | -2.36 | 0 (ref.) | -6.97 | -6.31 | -5.28 | -2.20 | 0.69 | 7.53 |  |
| Adjusted regression (% (95% CI)) | (-7.33;2.88) |  | (-11.25;-2.49) | (-10.48;-1.95) | (-9.22;-1.18) | (-7.00;2.85) | (-4.84;6.55) | (-0.06;15.71) | P<0.001 |
| Homocysteine (μmol/l) | 4.79 | 0 (ref.) | -1.79 | -1.79 | -3.72 | -3.22 | -1.57 | 4.90 |  |
| Adjusted regression (% (95% CI)) | (-0.77;10.66) |  | (-6.47;3.12) | (-6.32;2.97) | (-7.86;0.60) | (-8.36;2.21) | (-7.24;4.45) | (-2.99;13.43) | p=0.044 |

Data are β coefficients with 95% confidence intervals (CI) from adjusted regression analyses. β coefficients from models with log-transformed outcomes were back-transformed and reported as % with 95% CI. P values are F tests.
